# Adrenaline overdose in pediatric anaphylaxis: a case report

**DOI:** 10.1186/s13256-017-1290-7

**Published:** 2017-05-08

**Authors:** Pui Yi Lily Liew, John Andrew Craven

**Affiliations:** 1 Department of Emergency Medicine, Flinders Medical Centre, Flinders Drive, Bedford Park, 5042 Adelaide, Australia; 2 Present address: Department of Obstetrics and Gynaecology, Monash Health, Casey Hospital, 62-70 Kangan Drive, Berwick, 3806 Victoria Australia

**Keywords:** Pediatric, Children, Anaphylaxis, Adrenaline, Epinephrine, Overdose, Blood pressure, Case report

## Abstract

**Background:**

Adrenaline is the standard treatment for anaphylaxis but appropriate administration remains challenging, and iatrogenic overdose is easily overlooked. Despite the established importance of pediatric blood pressure measurement, its use remains inconsistent in clinical practice.

**Case presentation:**

We report a case of adrenaline overdose in a 9-year-old white boy with anaphylaxis, where signs of adrenaline overdose were indistinguishable from progressive shock until blood pressure measurement was taken.

**Conclusions:**

The consequences of under-dosing adrenaline in anaphylaxis are well-recognized, but the converse is less so. Blood pressure measurement should be a routine part of pediatric assessment as it is key to differentiating adrenaline overdose from anaphylactic shock.

## Background

Anaphylaxis, the most severe form of allergic reaction, is a life-threatening multi-systemic inflammatory process [1, 2] mediated by immunoglobulin E (IgE) [3]. The incidence of anaphylaxis in the pediatric group aged 5 to 14 has shown a marked upwards trend since 1990 [4, 5], and with the rapid onset and progression of the disease process, prompt action is key to successful management. Adrenaline is the recommended first line treatment in Australia, either through an autoinjector (commonly used in the community) or adrenaline ampoules and syringes [2]. Its use can be challenging due to non-intuitive dilution and dosing – particularly in the pediatric population.

We describe a case of adrenaline overdose in a child experiencing anaphylaxis, and discuss the role of blood pressure to differentiate progression of anaphylaxis symptoms from adrenaline toxicity.

## Case presentation

A 9-year-old white boy (weight 32 kg), known to have multiple allergies, asthma, and a history of anaphylaxis to dairy products and nuts, developed generalized body rash, dyspnea, and confusion after inadvertently ingesting a school meal containing a dairy product. He was promptly given 300 μg intramuscular adrenaline via a fixed-dose autoinjector, as well as 600 μg of salbutamol via metered-dose inhaler (six puffs) by his teacher in the community.

Upon ambulance arrival, he was drowsy, cyanosed, and had marked generalized body swelling. Observations suggested a shocked state: heart rate 120 beats/minute, respiratory rate 40 breaths/minute, air oxygen saturation 85 to 90%, loud respiratory wheeze, use of accessory muscles, and impalpable peripheral pulses. Further adrenaline was administered 300 μg intramuscularly without significant improvement, and this was followed by a bolus of 25 μg adrenaline administered intravenously. Twenty minutes into the event, on the presumption of refractory anaphylactic shock, an intravenous adrenaline infusion was commenced at 10 μg per minute. His blood pressure was not measured during the event or on the journey to the hospital.

In our emergency department, he was extremely agitated with repeated vomiting, but only had minimal respiratory distress. Oxygen saturation was difficult to record due to poor peripheral perfusion and clamminess, despite a blood pressure of 207/187 mmHg and heart rate of 160 beats/minute. Adrenaline toxicity was suspected and the adrenaline infusion was immediately ceased. He also received two separate doses of 2 mg intravenously administered ondansetron for the vomiting with little effect. Serial 12-lead electrocardiograms showed sinus tachycardia without other changes, and a normal chest X-ray excluded other causes.

Within 30 minutes his blood pressure returned to normal and he developed further respiratory symptoms (wheeze and dyspnea). The rapid improvement in his blood pressure, agitation, and vomiting following cessation of the adrenaline infusion supported the diagnosis of adrenaline overdose. In view of his asthma, he was given intravenously administered hydrocortisone and rescue nebulizers (salbutamol and ipratropium) to control his respiratory symptoms. He was admitted to our hospital, observed for 24 hours and discharged home with no further sequelae.

## Discussion

This case highlights adrenaline overdose, an iatrogenic complication rarely reported in the pediatric population, as a potential pitfall in the management of pediatric anaphylaxis. While delaying and under-dosing adrenaline in anaphylaxis is widely recognized as detrimental, one should remain vigilant for adrenaline overdose because the risk of adverse effects, such as ventricular arrhythmias, hypertensive crises, pulmonary edema, and associated mortality [1] is significant. Avoiding overdose in anaphylaxis requires careful attention to the administration of adrenaline, guided by the clinical status and vital signs of the patient.

Early recognition is pivotal in managing anaphylaxis. Children commonly demonstrate symptoms from one or more of the following systems: cutaneous (80 to 90%), respiratory (70%), gastrointestinal tract (45%), cardiovascular (45%), and central nervous (15%) [1]. Once anaphylaxis is diagnosed or suspected, adrenaline is the first line treatment [1, 2]. Australasian guidelines generally recommend administration of 10 μg/kg (up to 500 μg) of 1:1000 adrenaline as an intramuscular injection into the lateral thigh, repeated after 5 minutes if required [1, 2, 6]. When an adrenaline autoinjector is used, a 150 μg device is prescribed for children age 1 to 5 (approximately 10 to 20 kg) and 300 μg for children age 5 and above [2]. If the symptoms of anaphylaxis remain refractory, an intravenously administered adrenaline infusion can be started at the rate of 0.05 to 1 μg/kg per minute after careful consideration and consultation with emergency care specialists [2, 6].

Intramuscular injection is the standard route of administration. Skeletal muscle has an abundant supply of blood vessels and adrenaline is rapidly absorbed into the blood circulation [1, 2] but the risk of overdose and side effects are far lower compared with intravenously administered bolus or infusion. Adrenaline administered intravenously should only be administered in anaphylaxis if there is failure to respond to repeated intramuscular doses, or there is imminent or actual cardiorespiratory arrest [2, 6–8]. Other routes of administration are less reliable given the unpredictable absorption [9, 10].

Monitoring pediatric vital signs can sometimes be challenging. Blood pressure has always been recognized as a vital part of pediatric assessment but routine measurement remains inconsistent [11, 12]. In Australia, blood pressure measurement is recommended in the assessment of anaphylaxis in all age groups [2, 3, 6, 8] as it is the prime differentiator between anaphylactic shock and adrenaline overdose, as well as being an essential guide to anaphylaxis progression and need for further adrenaline dosing. Other manifestations of adrenaline toxicity such as tachycardia, palpitations, dyspnea, cold peripheries, agitation, and pallor [13] could be mistaken for worsening anaphylaxis, and further adrenaline may be incorrectly administered.

In cases of anaphylaxis where there is no apparent improvement with repeated adrenaline dosing, other differential diagnoses need be considered and/or further management needs to be initiated. Intravenously administered volume expansion should always be given in anaphylactic shock with repeated boluses of 20 mL/kg normal saline. Bronchodilators can be considered adjuncts in children with respiratory distress or a known history of asthma. Current evidence suggests no proven benefit for glucocorticoids or antihistamines in managing anaphylaxis [1, 6].

## Conclusions

Prompt adrenaline administration remains the cornerstone of managing anaphylaxis in the pediatric population; however, like all therapies it needs to be administered judiciously and its effect assessed to ensure correct dosing. In episodes of anaphylactic shock, blood pressure will provide a guide to the effectiveness of treatment and allows shock to be differentiated from adrenaline overdose.
